# Genetic Variants as Sudden-Death Risk Markers in Inherited Arrhythmogenic Syndromes: Personalized Genetic Interpretation

**DOI:** 10.3390/jcm9061866

**Published:** 2020-06-15

**Authors:** Oscar Campuzano, Georgia Sarquella-Brugada, Elena Arbelo, Sergi Cesar, Paloma Jordà, Alexandra Pérez-Serra, Rocío Toro, Josep Brugada, Ramon Brugada

**Affiliations:** 1 Cardiovascular Genetics Center, University of Girona-IDIBGI, 17190 Girona, Spain; aperez@gencardio.com; 2 Centro de Investigación Biomédica en Red. Enfermedades Cardiovasculares (CIBERCV), 28029 Madrid, Spain; elenaarbelo@secardiologia.es (E.A.); jbrugada@clinic.cat (J.B.); 3 Medical Science Department, School of Medicine, University of Girona, 17003 Girona, Spain; georgia@brugada.org; 4 Arrhythmias Unit, Hospital Sant Joan de Déu, University of Barcelona, 08950 Barcelona, Spain; sergi.cesar@gmail.com; 5 Arrhythmias Unit, Hospital Clinic, University of Barcelona-IDIBAPS, 08036 Barcelona, Spain; paloma.jorda.b@gmail.com; 6 Medicine Department, School of Medicine, 11003 Cadiz, Spain; rociotorogreen@gmail.com; 7 Cardiology Service, Hospital Josep Trueta, University of Girona, 17007 Girona, Spain

**Keywords:** sudden cardiac death, arrhythmias, next-generation sequencing, genetic diagnosis

## Abstract

Inherited arrhythmogenic syndromes are the primary cause of unexpected lethal cardiac episodes in young people. It is possible that the first sign of the condition may be sudden death. Inherited arrhythmogenic syndromes are caused by genetic defects that may be analyzed using different technical approaches. A genetic alteration may be used as a marker of risk for families who carry the genetic alterations. Therefore, the early identification of the responsible genetic defect may help the adoption of preventive therapeutic measures focused on reducing the risk of lethal arrhythmias. Here, we describe the use of massive sequencing technologies and the interpretation of genetic analyses in inherited arrhythmogenic syndromes.

## 1. Introduction

Cardiovascular diseases are the leading global cause of death, accounting for 30% of documented mortality (www.who.int/health-topics/cardiovascular-diseases). Sudden cardiac death (SCD) is responsible for most cardiovascular deaths, and coronary artery disease accounts for more than 80% of all SCD cases [1]. SCD accounts for 20% of deaths among young individuals, and results from familial genetic cardiomyopathies. Further, 5% to 10% of SCDs result from inherited arrhythmogenic syndromes (IASs) caused by channelopathies with alterations in ion channels or associated proteins [2]. IASs are usually autosomal-dominant, but autosomal-recessive, X-linked, and even mitochondrial-inheritance cases have been reported, and are usually associated with highly lethal episodes or syndromic phenotypes. Near Mendelian inheritance has been proposed, demonstrating a strong genetic factor modulated by additional genetic variants [3].

Channelopathies are IASs characterized by malignant electrical heart disturbances leading to ventricular fibrillation, syncope, and SCD. Because SCD is often the first sign of disease, early identification is critical to implement preventive measures. This is especially important in asymptomatic individuals for whom genetics are the only sign of risk. The four predominant IASs are long QT syndrome (LQTS), Brugada syndrome (BrS), catecholaminergic polymorphic ventricular tachycardia (CPVT), and short QT syndrome (SQTS) [4]. These inherited disorders are characterized by incomplete penetrance and variable expressivity, usually impeding definite diagnosis. Phenotypic overlap may be observed due to a combination of genetic variants and the additive effect of multiple independent variants [5]. Further, relatives from the same family can have different clinical presentations, from asymptomatic, to SCD with previous syncope, or even the first manifestation of disease without any previous symptoms. Initial phenotypic alterations may not be visible at autopsy, can be unspecific, or can be within the normal range [6]. Thus, almost 40% of SCD cases do not present cardiac anomalies after complete autopsy, and lethal IAS is often designated as the most plausible cause of death [7]. Clinical assessment and genetic analysis of relatives of an individual diagnosed with IAS is strongly recommended to determine the risk of malignant arrhythmia and SCD [8,9]. In this review, we discuss genetic approaches used to identify nonsynonymous variants in IAS and considerations for their interpretation.

## 2. Long QT Syndrome

LQTS is an IAS with an estimated prevalence of 1 in 2000. LQTS is characterized by electrocardiographically corrected QT (QTc) interval prolongation in the absence of a secondary cause for prolonged QTc, such as drugs or electrolyte disturbances. This arrhythmogenic disease is associated with ventricular arrhythmias, particularly “torsade des pointes”, leading to syncope and SCD. Further, LQTS is a common cause of sudden-infant-death syndrome [4]. LQTS can also be diagnosed in an individual with a risk score (modified Schwartz score) of >3.5 or upon identification of an unequivocally pathogenic variant in a LQTS-related gene.

**Figure 1:**
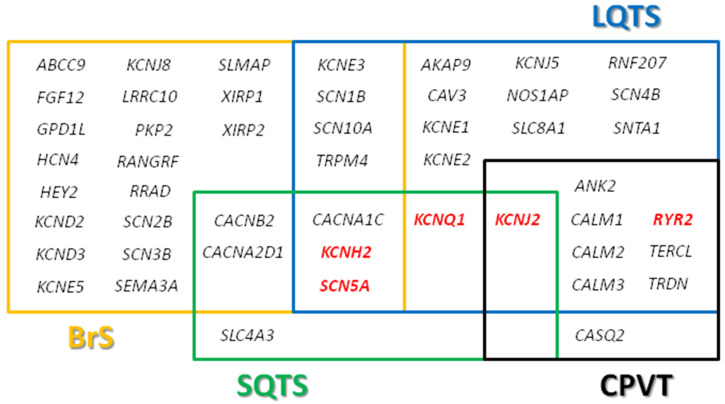
Genes associated with channelopathies. Main genes highlighted in red. BrS, Brugada syndrome; CPVT, catecholaminergic polymorphic ventricular tachycardia; LQTS, long QT syndrome; SQTS, short QT syndrome.

There are currently 26 genes associated with congenital LQTS (Figure 1), and comprehensive genetic analysis, including copy-number variants (CNV), identifies the genetic risk in nearly 90% of cases. However, more than 80% of cases are associated with rare nonsynonymous variants in genes encoding potassium or sodium ion channels (*KCNQ1*, *KCNH2*, and *SCN5A*). Current guidelines recommend analysis of only these three genes [10], and a recent international study concluded that only these three genes are linked to LQTS [11]. However, four other genes (*CALM1*, *CALM2*, *CALM3*, and *TRDN*) have strong causality for LQTS, but with atypical features such as sinus bradycardia or atrioventricular block, QT prolongation, seizures, or developmental delay in infancy or early childhood. Therefore, both congenital and acquired (typically drug-induced) LQTS represent distinct but intertwined arrhythmogenic disorders characterized by QT interval prolongation [12].

## 3. Brugada Syndrome

BrS is a rare IAS with a prevalence of 1 in 2500 characterized by electrocardiographic ST-segment elevation with successive negative T waves in at least one right precordial lead without structural cardiac abnormalities. A characteristic Type 1 Brugada pattern, observed spontaneously or induced during drug challenge, is considered definitively diagnostic. The most severe clinical symptom of BrS is ventricular fibrillation and SCD, which can be the first manifestation. Further, BrS is a main cause of SCD in children and young adults, although some patients remain asymptomatic for life [4]. Currently, 28 genes have been linked to BrS (Figure 1), and most follow an autosomal dominant pattern of inheritance, although some studies support autosomal recessive [13] or X-linked inheritance [14].

Comprehensive genetic analysis identifies genetic associations in nearly 35% of BrS cases, and up to 30% of genetic alterations are in *SCN5A.* Current guidelines recommend analysis of *SCN5A* as the most cost-effective approach [10]. *SCN5A* is considered pathogenic [15] despite only a few nonsynonymous variants that are considered deleterious [16]. Beyond *SCN5A*, pathogenic variants associated with BrS are in four minor genes: *SLMAP*, *SEMA3A*, *SCNN1A*, and *SCN2B* [17].

## 4. Catecholaminergic Polymorphic Ventricular Tachycardia

CPVT is a rare (prevalence of 1 in 10,000) highly lethal IAS with a 30% mortality rate in untreated patients. It is characterized by adrenergically stimulated polymorphic ventricular tachycardia in the presence of a structurally normal heart. CPVT is usually diagnosed in patients younger than 40 years old [18]. The diagnostic hallmark is induced ventricular arrhythmias during exercise, particularly bidirectional ventricular tachycardia. A key feature of CPVT is a normal baseline electrocardiogram and echocardiogram. Without exercise stress testing, diagnosis can be missed [19].

Nine genes are associated with CPVT (Figure 1), and genetic alteration (noncommon variants and CNV) is a potential cause in almost 65% of cases, although 60% of cases are attributed to rare nonsynonymous variants in the cardiac ryanodine receptor (*RYR2*) [20]. Current guidelines recommend analysis of *RYR2* in CPVT diagnosis [10]. Further, a recent international calmodulinopathy registry identified that nearly 28% of patients diagnosed with CPVT had alterations in calmodulin genes (mainly *CALM2*). All CALM–CPVT patients were symptomatic with early age of onset (around 6 years old) [21]. Identification of a pathogenic variant implies that genetic testing should be extended to first-degree relatives since CPVT is highly lethal.

## 5. Short QT Syndrome

Short QT syndrome (SQTS) is rare, with a prevalence of 1 in 10,000. SQTS is associated with paroxysmal atrial and ventricular fibrillation, syncope, and SCD, and is characterized by a short QT interval on the electrocardiogram, lack of normal changes in QT interval with heart rate, peaked T waves (particularly in precordial leads), and short or absent ST segments. The most common initial symptom is cardiac arrest in one-third of cases. Lethal episodes usually occur in infants and young children with no structural heart abnormalities [22]. SQTS is a genetically heterogeneous disease with eight associated genes (Figure 1). Comprehensive genetic analysis identified a genetic cause in 40% of cases, with most diagnosed cases resulting from alterations in *KCNH2*, *KCNQ1*, and *KCNJ2*. Current guidelines recommend analysis of these three genes [10]. Our group recently reported that rare variants in other genes are associated with electrical alterations concomitant with shortened QT intervals, but do not guarantee a diagnosis of SQTS [23]. Thus, other genetic alterations may explain cases without definitive genetic diagnosis. Additional large studies are needed, but low prevalence and high mortality rates impede comprehensive genotype–phenotype studies.

## 6. Genetic Diagnosis

**Figure 2:**
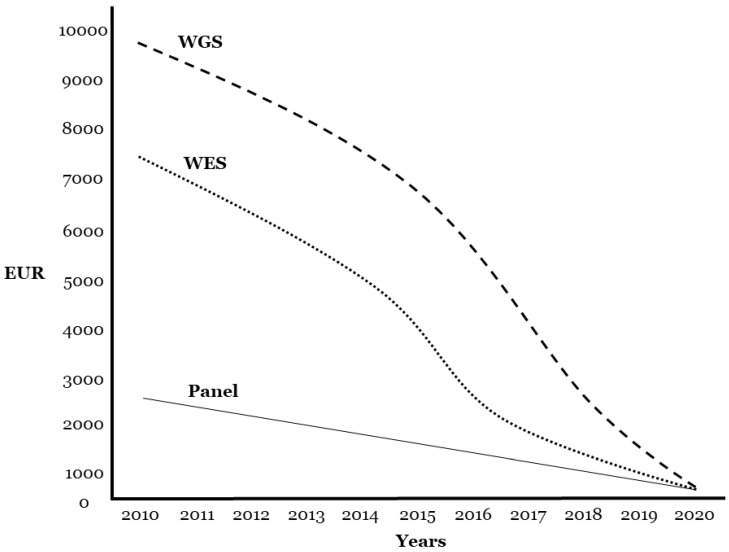
Cost for different next-generation-sequencing (NGS) approaches. Approximate price in euros per sample for WGS, WES, and targeted gene panels in last ten years. EUR, euros; WES, whole-exome sequencing; WGS, whole-genome sequencing.

First, it is important to remember that the human-genome sequence was obtained nearly 20 years ago at an estimated cost of $3 billion. Next-generation sequencing (NGS) has changed the landscape of genetic testing in the past 10 years, and introduced newer, faster, and cheaper genetic sequencing. However, the rapid evolution of genetic screening has outpaced its clinical translation [24]. Current genetic technology facilitates comprehensive analysis of all genes associated with IAS using a resequencing panel. The current price of a panel is EUR 1500 or less, depending on the inclusion of genetic interpretation, and the cost is similar if one large (>50 exons) or two medium genes (25 exons) are amplified using Sanger technology. For a similar price, whole-exome sequencing (WES) or whole-genome sequencing (WGS) can be used (Figure 2). In addition, new genetic technology can perform WGS for USD 500, albeit without genetic interpretation, with expected progressive cost reduction (www.technologyreview.com/s/615289/china-bgi-100-dollar-genome).

Despite the availability of WES/WGS, it is important to consider that massive genetic analysis implies the identification of many rare variants in unknown genes not related to clinical diagnosis, as well as false-positive variants due to low coverage [25]. Further, the genetic interpretation and clinical translation of results are challenging, as most genetic variants remain of unknown/ambiguous significance. Identification of incidental findings derived from WES/WGS is well-accepted, and what to do with them is a current matter of discussion. It has been recommended that at least results from 59 genes (definitely associated with severe diseases), should be returned in clinical genomic sequencing [26]. The impact of variants in noncoding regions is not well-understood despite the influence on expression and mRNA splicing affecting protein abundance and isoforms. Following Mayo Clinic recommendations, WES is currently recommended for analysis of patients with negative results in conventional genetic tests or panel-based NGS, and provides a potentially cost-effective alternative to establishing molecular diagnosis compared to multiple independent molecular assays (www.mayocliniclabs.com/test-catalog/Overview/64580). Further, WES combined with phenotype-driven gene lists (virtual panel) will soon be the most appropriate approach, offering the advantage of reanalyzing new genes discovered in the near future [27].

Despite having similar cost, the genetic yield of analyzing five genes associated with IAS (*KCNQ1*, *KCNH2*, *SCN5A*, *KCNJ2*, and *RyR2*) is similar to that in large panels, including more than 100 genes [28]. Therefore, current guidelines for IAS recommend analysis of these five genes [10], although every year, new genes are associated with IAS. Further, recent studies showed that larger panels, including genes with limited association with IAS, provided minimal diagnostic yield, but increased the detection of variants classified with ambiguous clinical significance (variant of unknown significance, VUS) [11,17]. Therefore, careful interpretation of genetic tests is critical to appropriately care for patients and their relatives.

## 7. Genetic Translation

American College of Medical Genetics and Genomics and the Association for Molecular Pathology (ACMG/AMP) guidelines classify all variants associated with arrhythmogenic diseases and SCD [29]. However, more than 50% of rare variants recently changed classification according to updated ACMG guidelines due to modifications performed in the current guidelines [30,31]. Genetic alterations with potential deleterious roles, classified as pathogenic (P) or likely pathogenic (LP), in an individual diagnosed with IAS, allow familial screening via cascade testing to identify at-risk individuals. Reporting of P and LP variants should thus be accompanied by functional data as proof of accuracy despite, existing limitations that should be considered [32]. Ultimately, functional analysis of IAS variants is an important but not definitive tool due to variants that often remain ambiguous after classification following ACMG guidelines. Therefore, different tools such as in silico predictions may help clarify the variants’ roles [32,33]. Functional studies can also help clarify the role of each VUS, but are not routinely performed. Family segregation is the most important information required to unravel the actual role of a VUS in each family member. In families with multiple affected individuals, genetic testing for the VUS in other affected relatives may help clarification by determining whether the variant segregates with disease. Therefore, an update in the current guidelines should be published in order to clarify several items that still remain unclear.

A VUS is an inconclusive result when there is either insufficient or conflicting evidence regarding a variant’s pathogenicity. A VUS does not provide the molecular confirmation of a diagnosis, but it cannot be discarded. Therefore, families should be counseled regarding the limits of the current ability to provide reliable clinical interpretation. Further, both clinicians and patients should be aware that a variant may be reclassified as additional evidence accumulates [16,34]. In the future, periodic re-evaluation of rare variants, especially if previously classified as VUS, should be performed for IAS assuming clinical consequences.

In previous years, common variants in IAS were proposed to have a role as phenotype modifiers [35], and more recently as potential causative variants in association with rare alterations. Therefore, a single rare variant may not be enough to cause the phenotype due to the increasing number of common variants now thought to be clinically relevant. Thus, an oligogenic model should replace the traditional Mendelian model for BrS [36]. It was also suggested that the only potential role of common variants was as causative agents in IAS [37], despite the lack of definite data concerning this point. Recently, it was demonstrated that a single synonymous codon substitution may alter a protein-folding mechanism in vivo, leading to changes in cellular fitness causing a disease [38]. Hence, common variants also underlie susceptibility to drug-induced QT prolongation, but do not cause overt LQTS [12]. As in LQTS, common variants have been suggested to shorten the QT interval, but no common variants have been reported as being definitely causative. Further, few drugs are associated with the QT interval [39]. Despite these facts, large studies are needed to clarify the role of synonymous variants in IAS. Finally, it is important to mention that due to the low genetic yield after comprehensive genetic analysis focused on Mendelian inheritance in exonic regions, alternative genetic analysis of regulatory regions has also been performed in IAS, such as in BrS [40]. WGS is the proper approach to analyze these regions, but subsequent functional interpretation using in vitro and in vivo models is needed before clinical translation.

## 8. Technical Data

Technical and bioinformatic data are crucial in NGS analysis. The filtration and annotation of genetic variants include multiple components, and the diagnostic decision depends on the data resources used. Recent studies recommended NGS coverage of at least 20× for diagnostic purposes [41]. Despite the high coverage obtained using gene panels, Sanger technology should be used to confirm all variants included in a genetic report and to discard false positives, especially concerning insertions/deletions (indels). In clinical diagnosis, all exons included in a genetic report with coverage of less than 30× (included, not covered) should be amplified using Sanger sequencing. If not analyzed, these exons should be specified in the genetic report, as any rare variants associated with disease could exist in incorrectly amplified regions. There is an increased rate of false positives in areas not sufficiently amplified [25]. Thus, Sanger confirmation is important in all DNA extracted from blood, saliva, or fresh/frozen tissue, and crucial in DNA extracted from formalin-fixed paraffin-embedded tissue (FFPE) [42]. In DNA from FFPE fixed for more than 8 days, high ratios of both false positives and false negatives were identified, and reanalysis of samples obtained from different areas of the FFPE block is recommended.

**Figure 3:**
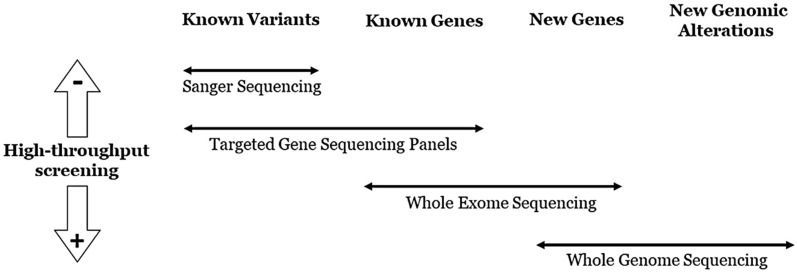
Use of different NGS approaches.

Technology focused on genetic panels usually obtains high coverage. This improvement allows the bioinformatic identification of other structural alterations such as rearrangements and CNVs, which are associated with 2–10% of diagnosed IASs [43,44]. However, WES does not provide enough coverage for exhaustive CNV analysis. Alternative techniques such as multiplex ligation-dependent probe amplification (MLPA) or real-time polymerase chain reaction (rtPCR) should be used to identify CNVs after WES, or to confirm CNVs identified in gene panels. A similar situation occurs in WGS, but with lower coverage. A genetic report including WES or WGS results from families with IAS has yet to be established [43] despite that, in the near future, both genetic approaches will be the main technologies in genetic diagnosis (Figure 3). Nonetheless, panels encompassing all genes associated with IAS are the best approach for the genetic diagnosis of both living patients and postmortem cases of unexplained sudden decease with suspected arrhythmia.

## 9. Conclusions

Nowadays, different approaches are available to perform genetic analyses. IAS may lead to sudden death, sometimes as the first symptom of the disease. Identification of genetic defects may be used as markers of patients at risk. The characterization of genetic factors associated with IAS and the proper clinical translation of genetic data are crucial for clinical diagnosis and adoption of personalized therapeutic measures. Further, early identification of genetic alterations in family members may prevent lethal episodes, especially in asymptomatic individuals. Appropriate genetic testing should be used in each case, focused on the identification of definite genetic variants associated with the diagnosed clinical condition. This proper approach provides informative genetic data in diagnosis. Personalized interpretation should be done in multidisciplinary centers specialized in cardiovascular genetics.

## References

[1] Zipes D.P., Wellens H.J. (1998). Sudden cardiac death. Circulation.

[2] Bagnall R.D., Weintraub R.G., Ingles J., Duflou J., Yeates L., Lam L., Davis A.M., Thompson T., Connell V., Wallace J. (2016). A Prospective Study of Sudden Cardiac Death among Children and Young Adults. N. Engl. J. Med..

[3] Bezzina C.R., Lahrouchi N., Silvia P.G. (2015). Genetics of Sudden Cardiac Death. Circ. Res..

[4] Singh M., Morin D.P., Link M.S. (2019). Sudden cardiac death in Long QT syndrome (LQTS), Brugada syndrome, and catecholaminergic polymorphic ventricular tachycardia (CPVT). Prog. Cardiovasc. Dis..

[5] Coll M., Perez-Serra A., Mates J., Del Olmo B., Puigmulé M., Fernandez-Falgueras A., Iglesias A., Pico F., Lopez L., Brugada R. (2017). Incomplete Penetrance and Variable Expressivity: Hallmarks in Channelopathies Associated with Sudden Cardiac Death. Biology.

[6] Christensen N.L., Carter-Storch R., Bakkestrøm R., Dahl J.S. (2016). Sudden cardiac death in asymptomatic aortic stenosis: Is the valve to blame?. BMJ Case Rep..

[7] Neubauer J., Lecca M.R., Russo G., Bartsch C., Medeiros-Domingo A., Berger W., Haas C. (2017). Post-mortem whole-exome analysis in a large sudden infant death syndrome cohort with a focus on cardiovascular and metabolic genetic diseases. Eur. J. Hum. Genet..

[8] Hershberger R.E., Givertz M.M., Ho C.Y., Judge D.P., Kantor P., McBride K.L., Morales A., Taylor M.R., Vatta M., Ware S.M. (2018). Genetic Evaluation of Cardiomyopathy—A Heart Failure Society of America Practice Guideline. J. Card. Fail..

[9] Hershberger R.E., Givertz M.M., Ho C.Y., Judge D.P., Kantor P.F., McBride K.L., Morales A., Taylor M.R.G., Vatta M., Ware S.M. (2018). Genetic evaluation of cardiomyopathy: A clinical practice resource of the American College of Medical Genetics and Genomics (ACMG). Genet. Med..

[10] Priori S.G., Blomström-Lundqvist C. (2015). CardioPulse Articles2015 European Society of Cardiology Guidelines for the management of patients with ventricular arrhythmias and the prevention of sudden cardiac death summarized by co-chairs ‘Ten Commandments’ of 2015 European Society of Cardiology Guidelines for the management of patients with ventricular arrhythmias and the prevention of sudden cardiac deathAdolfo J. de Bold PhD OC FRSC: A pioneer in cardiovascular medicineNatriuretic peptides in 2015Teachable moment or missed opportunity?. Eur. Hear. J..

[11] Adler A., Novelli V., Amin A.S., Abiusi E., Care M., Nannenberg E.A., Feilotter H., Amenta S., Mazza D., Bikker H. (2020). An International, Multicentered, Evidence-Based Reappraisal of Genes Reported to Cause Congenital Long QT Syndrome. Circulation.

[12] Giudicessi J.R., Roden D.M., Wilde A.A., Ackerman M.J. (2018). Classification and Reporting of Potentially Proarrhythmic Common Genetic Variation in Long QT Syndrome Genetic Testing. Circulation.

[13] Janin A., Bessière F., Georgescu T., Chanavat V., Chevalier P., Millat G. (2019). TRPM4 mutations to cause autosomal recessive and not autosomal dominant Brugada type 1 syndrome. Eur. J. Med. Genet..

[14] David J.P., Lisewski U., Crump S.M., Jepps T.A., Bocksteins E., Wilck N., Abbott G.W. (2019). Deletion in mice of X-linked, Brugada syndrome- and atrial fibrillation-associated Kcne5 augments ventricular KV currents and predisposes to ventricular arrhythmia. FASEB J..

[15] Hosseini S.M., Kim R., Udupa S., Costain G., Jobling R., Liston E., Jamal S.M., Szybowska M., Morel C.F., Bowdin S. (2018). Reappraisal of Reported Genes for Sudden Arrhythmic Death. Circulation.

[16] Denham N., Pearman C.M., Ding W.Y., Waktare J., Gupta D., Snowdon R., Hall M., Cooper R., Modi S., Todd D. (2018). Systematic re-evaluation of SCN5A variants associated with Brugada syndrome. J. Cardiovasc. Electrophysiol..

[17] Campuzano O., Sarquella-Brugada G., Fernandez-Falgueras A., Cesar S., Coll M., Mates J., Arbelo E., Perez-Serra A., Del Olmo B., Jordá P. (2019). Genetic interpretation and clinical translation of minor genes related to Brugada syndrome. Hum. Mutat..

[18] Kim C.W., Aronow W.S., Dutta T., Frenkel D., Frishman W.H. (2020). Catecholaminergic Polymorphic Ventricular Tachycardia. Cardiol. Rev..

[19] Refaat M.M., Hotait M., Tseng Z.H. (2014). Utility of the Exercise Electrocardiogram Testing in Sudden Cardiac Death Risk Stratification. Ann. Noninvasive Electrocardiol..

[20] Wleklinski M.J., Kannankeril P.J., Knollmann B.C. (2020). Molecular and tissue mechanisms of catecholaminergic polymorphic ventricular tachycardia. J. Physiol..

[21] Crotti L., Spazzolini C., Tester D.J., Ghidoni A., Baruteau A.-E., Beckmann B.-M., Behr E.R., Bennett J., Bezzina C.R., A Bhuiyan Z. (2019). Calmodulin mutations and life-threatening cardiac arrhythmias: Insights from the International Calmodulinopathy Registry. Eur. Hear. J..

[22] Campuzano O., Sarquella-Brugada G., Cesar S., Arbelo E., Brugada J., Brugada R. (2018). Recent Advances in Short QT Syndrome. Front. Cardiovasc. Med..

[23] Campuzano O., Fernandez-Falgueras A., Maulen X.L., Sarquella-Brugada G., César S., Coll M., Mates J., Arbelo E., Jordà P., Perez-Serra A. (2019). Short QT Syndrome: A Comprehensive Genetic Interpretation and Clinical Translation of Rare Variants. J. Clin. Med..

[24] Liu R., Zhang P. (2019). Towards early detection of adverse drug reactions: Combining pre-clinical drug structures and post-market safety reports. BMC Med. Inform. Decis. Mak..

[25] Schwarze K., Buchanan J., Taylor J.C., Wordsworth S. (2018). Are whole-exome and whole-genome sequencing approaches cost-effective? A systematic review of the literature. Genet. Med..

[26] Kalia S.S., Adelman K., Bale S.J., Chung W.K., Eng C., Evans J.P., Herman G.E., Hufnagel S.B., Klein T.E., on behalf of the ACMG Secondary Findings Maintenance Working Group (2016). Recommendations for reporting of secondary findings in clinical exome and genome sequencing, 2016 update (ACMG SF v2.0): A policy statement of the American College of Medical Genetics and Genomics. Genet. Med..

[27] Dillon O.J., Lunke S., Stark Z., Yeung A., Thorne N., Gaff C., White S.M., Tan T.Y., Melbourne Genomics Health Alliance (2018). Exome sequencing has higher diagnostic yield compared to simulated disease-specific panels in children with suspected monogenic disorders. Eur. J. Hum. Genet..

[28] Marschall C., Moscu-Gregor A., Klein H.-G. (2019). Variant panorama in 1385 index patients and sensitivity of expanded next-generation sequencing panels in arrhythmogenic disorders. Cardiovasc. Diagn. Ther..

[29] Richards S., Aziz N., Bale S., Bick D., Das S., Gastier-Foster J., Voelkerding K. (2018). Faculty Opinions recommendation of Standards and guidelines for the interpretation of sequence variants: A joint consensus recommendation of the American College of Medical Genetics and Genomics and the Association for Molecular Pathology. Fac. Opin..

[30] Bennett J.S., Bernhardt M., McBride K.L., Reshmi S.C., Zmuda E., Kertesz N.J., Garg V., Fitzgerald-Butt S., Kamp A.N. (2019). Reclassification of Variants of Uncertain Significance in Children with Inherited Arrhythmia Syndromes is Predicted by Clinical Factors. Pediatr. Cardiol..

[31] Campuzano O., Sarquella-Brugada G., Fernandez-Falgueras A., Coll M., Iglesias A., Ferrer-Costa C., Cesar S., Arbelo E., García-Álvarez A., Jordà P. (2020). Reanalysis and reclassification of rare genetic variants associated with inherited arrhythmogenic syndromes. EBioMedicine.

[32] Gando I., Yang H.-Q., Coetzee W.A. (2018). Functional significance of channelopathy gene variants in unexplained death. Forensic Sci. Med. Pathol..

[33] Riuró H., Campuzano O., Berne P., Arbelo E., Iglesias A., Pérez-Serra A., Coll-Vidal M., Partemi S., Mademont-Soler I., Picó F. (2014). Genetic analysis, in silico prediction, and family segregation in long QT syndrome. Eur. J. Hum. Genet..

[34] Olubando D., Hopton C., Eden J., Caswell R., Thomas N.L., Roberts S.A., Morris-Rosendahl D., Venetucci L., Newman W. (2020). Classification and correlation of RYR2 missense variants in individuals with catecholaminergic polymorphic ventricular tachycardia reveals phenotypic relationships. J. Hum. Genet..

[35] Jenewein T., Neumann T., Erkapic D., Kuniss M., Verhoff M.A., Thiel G., Kauferstein S. (2017). Influence of genetic modifiers on sudden cardiac death cases. Int. J. Leg. Med..

[36] Cerrone M., Remme C.A., Tadros R., Bezzina C.R., Delmar M. (2019). Beyond the One Gene-One Disease Paradigm: Complex Genetics and Pleiotropy in Inheritable Cardiac Disorders. Circulation.

[37] Behr E.R., Savio-Galimberti E., Barc J., Holst A.G., Petropoulou E., Prins B., Jabbari J., Torchio M., Berthet M., Mizusawa Y. (2015). Role of common and rare variants in SCN10A: Results from the Brugada syndrome QRS locus gene discovery collaborative study. Cardiovasc. Res..

[38] Walsh I.M., Bowman M.A., Santarriaga I.F.S., Rodriguez A., Clark P.L. (2020). Synonymous codon substitutions perturb cotranslational protein folding in vivo and impair cell fitness. Proc. Natl. Acad. Sci. USA.

[39] Malik M. (2016). Drug-Induced QT/QTc Interval Shortening: Lessons from Drug-Induced QT/QTc Prolongation. Drug Saf..

[40] Daimi H., Khelil A.H., Neji A., Ben Hamda K., Maaoui S., Aranega A., Chibani J.B., Franco D. (2019). Role of SCN5A coding and non-coding sequences in Brugada syndrome onset: What’s behind the scenes?. Biomed. J..

[41] Hartman P., Beckman K., Silverstein K., Yohe S., Schomaker M., Henzler C., Onsongo G., Lam H.C., Munro S., Daniel J. (2019). Next generation sequencing for clinical diagnostics: Five year experience of an academic laboratory. Mol. Genet. Metab. Rep..

[42] Lin Y., Gryazeva T., Wang D., Zhou B., Um S.Y., Eng L.S., Ruiter K., Rojas L., Williams N., Sampson B.A. (2020). Using postmortem formalin fixed paraffin-embedded tissues for molecular testing of sudden cardiac death: A cautionary tale of utility and limitations. Forensic Sci. Int..

[43] Mates J., Mademont-Soler I., Fernandez-Falgueras A., Sarquella-Brugada G., Cesar S., Arbelo E., Fiol V. (2020). Sudden Cardiac Death and Copy Number Variants: What Do We Know after 10 Years of Genetic Analysis?. Forensic Sci. Int. Genet..

[44] Mademont-Soler I., Pinsach-Abuin M., Riuró H., Matés J., Pérez-Serra A., Coll M., Porres J.M., Del Olmo B., Iglesias A., Selga E. (2016). Large Genomic Imbalances in Brugada Syndrome. PLoS ONE.

